# Preoperative Rehearsal in the Removal of an Airway Foreign Body in a Preterm Septic Neonate

**DOI:** 10.1155/crot/8812622

**Published:** 2025-05-04

**Authors:** Nikhil Bellamkonda, J. Fredrik Grimmer

**Affiliations:** Department of Otolaryngology, Head and Neck Surgery, University of Utah, Salt Lake City, Utah, USA

**Keywords:** catheter foreign body, foreign body, neonatal foreign body, preoperative planning

## Abstract

We report a case of a septic, 21 day old, former 26-week neonate who had clinical and x-ray concern for an airway foreign body. 3D CT remodeling was used to identify the foreign body as the tip of a suction catheter. Preoperative planning to confirm optimal bronchoscopic instrumentation was done, and the foreign body was successfully removed in a single attempt. This case highlights the importance of preoperative radiographic evaluation and instrument rehearsal in high-risk airway foreign body cases.

## 1. Introduction

Foreign body aspiration (FBA) is a major cause of morbidity and mortality in children and is a common cause of accidental death. It is a frequent presentation seen by pediatric otolaryngologists and is treated as an emergent condition [1].

FBA most frequently occurs in children under the age of three. Neonatal foreign bodies, however, are exceedingly rare and are often iatrogenic in nature. These cases are unique, in that patients are often intubated, have pre-existing pulmonary derangements, bronchopulmonary dysplasia, and are unable to display symptoms classically associated with FBA.

There have been several cases of iatrogenic airway foreign body previously published in the literature [2–4]. Here, we present a case of a retained iatrogenic airway foreign body in a septic, three-week-old, premature neonate, with demonstration of preoperative planning that led to successful outcome.

## 2. Case Presentation

Patient was born at 26 6/7 weeks to a mother with a history of fetal loss due to cervical incompetence, who presented after premature rupture of membranes. Patient received CPAP briefly and then was advanced to positive pressure ventilation. Several attempts were required before a successful intubation was achieved.

At 1 week of life, patient was briefly weaned and then reintubated with a larger endotracheal tube (ETT). At this time, there was blood suctioned from the ETT, despite atraumatic intubation. The bleeding cleared after several hours. Patient continued to require high frequency oscillatory ventilation. On Day 11 of life, they had worsening secretions, and tracheal aspirate grew multidrug resistant *Klebsiella*. They were continued on broad spectrum antibiotics.

**Figure 1:**
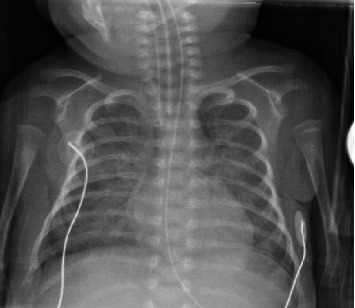
AP chest x-ray initially revealing foreign body within the left mainstem bronchus.

**Figure 2:**
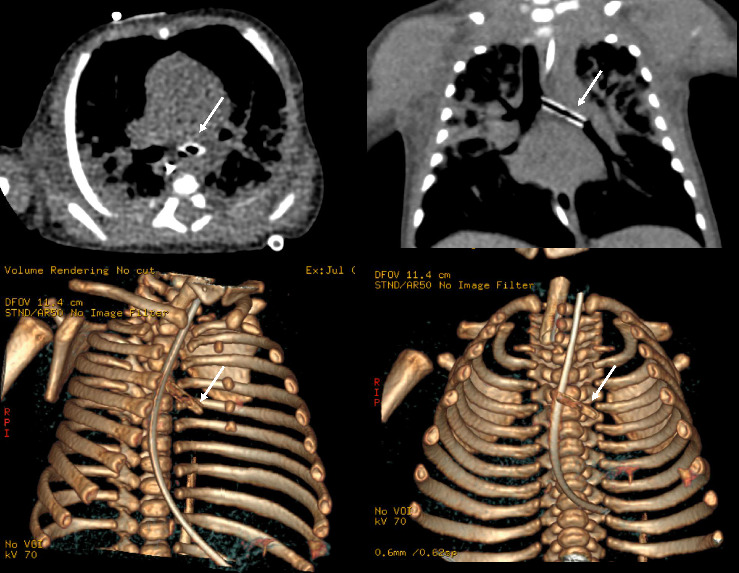
CT scan with 3D remodeling. White arrow indicates cylindrical foreign body.

At around 3 weeks of life, patient developed increasing respiratory distress. A chest x-ray at this time revealed a left-sided cylindrical density over the left mediastinum/mainstem bronchus (Figure 1) that was also visible on CXR done within the last 24 h. Initially, this was considered to be external to the infant and artifactual. However, a similar density was then noted on CXR from 48 h prior. The patient was then transferred to our facility for higher level care. Upon arrival, a chest CT was completed and confirmed a 14 mm length, 2.8 mm diameter, cylindrical foreign body within the left mainstem bronchus (Figure 2).

**Figure 3:**
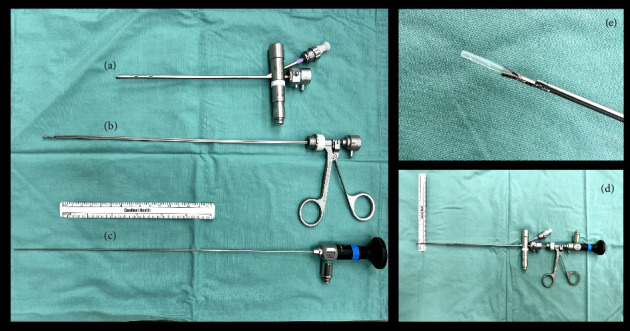
(a) Size 2.5 bronchoscope. (b) Miniature optical forceps. (c) 1.9 mm 0 degree telescope. (d) Instruments combined. (e) Grasping a 5-0 catheter tip.

It was decided that the patient would require emergent evaluation in the OR for removal. This was complicated by several factors; primarily that patient was septic secondary to this pneumonia and that ECMO was not an option due to their age [5, 6]. We conducted a preoperative simulation to maximize chance of success at first attempt. Given the patient's age, a size 2.5 bronchoscope was selected. We determined based on the CT scan and 3D remodeling that a suction catheter tip was the most likely foreign body culprit. Various instruments were trialed to determine optimal grasping of a similarly sized catheter. The Karl Storz miniature optical forceps with alligator jaws was selected and paired with a 1.9 mm 0° rigid endoscope (Figure 3).

In the operating room, the patient was preoxygenated and then extubated. The larynx was exposed and a size 2.5 ventilating bronchoscope was inserted and advanced to the left mainstem bronchus, with the foreign body clearly in view. The optical alligator grasper was inserted. The proximal edge of the foreign body was grasped firmly. The bronchoscope and grasper were then withdrawn in unison, taking care not to release the foreign body. The patient was quickly reintubated upon successful removal. The foreign body was confirmed to be the cut tip of a 5-0 French Ballard suction catheter.

## 3. Discussion

This case demonstrates the utility of preoperative planning and simulation in critically ill pediatric patients with an airway foreign body. Various other innovative methods of instrumentation have been described in the literature; from attempts at suctioning through the ETT, blind grasping while using precise distance measurements, or use of nontraditional instrument such as sialoendoscopy or urologic instruments [7–10].

In patients with chronic respiratory derangement rather than acute decompensation, tactical and multidisciplinary planning is the key to success. Appropriate imaging to identify characteristics of the foreign body can allow for selection of proper equipment. Treatment with antibiotics, anti-inflammatories, and bronchodilators to optimize pulmonary function should be pursued prior to movement to the operating room.

While creativity in instrumentation can be fruitful, preoperative rehearsal and simulation is of critical importance. Many of these children have poorly pulmonary reserve and are too young to be considered for ECMO in case of failure. In this case, given the preoperative imaging appearance and 3D CT remodeling, we were able to predict the etiology of the foreign body. This allowed for precise instrument selection by trialing which tool would best grasp an identical suction catheter piece. Doing so facilitated grasping and removal of the suction catheter in a single attempt. Another previously published report with a similar patient scenario discussed usage of flexible bronchoscopy through an ETT to identify the foreign body, while maintaining airway access [11].

At our center, we have multiple dedicated pediatric foreign body instrument carts, which facilitate efficient instrument selection. Within this, we stock the Karl Storz Miniature Optical Forceps with alligator jaws (KS 10374 HF), which proves highly useful for delicate cases like this one.

Prevention methods are also critical. In preterm neonates, there have been prior reports of cut suction catheter foreign bodies, as well as sheared tips of stylets used in intubation [12, 13]. Suction catheter airway foreign bodies can occur if the proximal end of the ETT is trimmed by a respiratory therapist or physician while the suction catheter is accidentally left in place. At our institution, this event resulted in a quality assurance review; the practice of ETT trimming is now highly discouraged, and if necessary is only permitted when an attending physician is present [14, 15]. Leung et al. recommended that manufacturers add colored graduation marks to the distal ends of suction catheters, which would facilitate confirmation of intactness of the catheter [12].

## 4. Conclusion

Extraction of an airway foreign body from a preterm, septic neonate can be done safely with proper preoperative planning. The 3D remodeling of CT scans to better identify origin and location of the foreign body and operative simulation drills are the key to success and patient safety.

## Data Availability

Data sharing is not applicable to this article as no new data were created or analyzed in this study.
